# The Effect of Simultaneous Contralateral White Noise Masking on Cortical Auditory Evoked Potentials Elicited by Speech Stimuli

**DOI:** 10.1055/s-0043-1767675

**Published:** 2024-02-05

**Authors:** Luiza Dandara de Araújo Felix, Pedro Lemos Menezes, Lisiane Vital de Oliveira, Carlos Henrique Alves Batista, Aline Tenório Lins Carnaúba, Kelly Cristina Lira de Andrade

**Affiliations:** 1 Centro Universitário CESMAC, Maceió, AL, Brazil; 2 Universidade Estadual de Ciências da Saúde de Alagoas, Maceió, AL, Brazil; 3 Universidade Estadual de Ciências da Saúde de Alagoas, Residency in Audiology, Maceió, AL, Brazil

**Keywords:** electrophysiology, auditory evoked potentials, noise

## Abstract

**Introduction** Noise obscures speech signal, causing auditory masking. The effects of this masking can be observed through the cortical auditory evoked potentials (CAEPs). White noise, in turn, has an effect on the auditory cortex, interfering, for example, with lexical decision making.

**Objective** To analyze the effect of simultaneous masking by contralateral white noise on CAEPs elicited by speech stimuli.

**Methods** Cross-sectional observational analytical study carried out with 15 participants of both sexes, who were submitted to CAEPs in two conditions: 1) without noise; 2) with white noise at 100 dBSPL intensity, contralaterally and simultaneously. To compare these conditions, the Student t test or the Wilcoxon test were used, depending on the sample normality. Differences with p values < 0.05 were considered significant.

**Results** : When white noise was presented contralaterally and simultaneously to the CAEPs with speech stimulus, an increase in P1, N1 and P2 wave latencies was observed. P1 and P2 amplitudes and N1-P2 peak to peak amplitude also increased, unlike N1 amplitude, which decreased. The differences were significant for P1 and P2 wave latencies and for P2 wave amplitude.

**Conclusion** The simultaneous masking effect was observed from the morphological alterations of the CAEPs with speech stimulus when white noise was presented in the contralateral ear. There was a significant increase in P1 and P2 wave latencies, as well as in P2 wave amplitude.

## Introduction

Speech discrimination depends on intrinsic and extrinsic aspects such as the acoustic characteristics of the speech signal and external noise. 1 As noise is present in distinct everyday environments, listeners are constantly challenged to filter it to discriminate and understand speech. This effort is necessary because noise obscures the less intense portions of the speech signal, thus configuring auditory masking. 2

Auditory masking is understood as decreased sound (signal) audibility due to the presence of another sound (masker). 3 Auditory masking can occur at any level of the auditory pathway, from the cochlea to the cortex, and is caused not only when the masker is presented to the ipsilateral ear, but also when the masker is presented to the contralateral ear. 4

When the masker is presented to the ipsilateral ear, peripheral auditory masking occurs. The spectral overlap between the signal and the mask causes interference from the cochlea. When the masker is presented to the contralateral ear, central auditory masking occurs. The spectral overlap between the signal and the masker causes interference at the cortical level. 5

Auditory masking can be observed through electrophysiological tests, such as auditory evoked potentials (AEPs). Studies have shown that masking weakens and prolongs neural processing of speech. AEPs also aid to determine, for instance, whether the difficulties presented by the individual are related to abnormal neural processing of speech or to cognitive conditions. 6 7

The cortical auditory evoked potentials (CAEPs) seem to be the most promising examination for assessing the neural processing of speech, since they reflect the synchronous activity of the structures in the thalamocortical segment of the central auditory system. 5 Its responses consist of a complex of three waves (P1-N1-P2) elicited by different acoustic stimuli and recorded without the active participation of the examined individual. 7

Cortical auditory evoked potentials can be elicited with different acoustic stimuli, such as pure tone, speech, and noise. In auditory masking studies, masking can be presented simultaneously (simultaneous masking), before (premasking) and after (postmasking) the signal. 8 Noise (masker) is believed to affect CAEP responses with speech stimuli (signal) regardless of their position, increasing their latencies and decreasing their amplitudes 8 9 10 11 12 13 14 15 .

How noise affects CAEP responses depends on several factors, especially the type of masking and its intensity in relation to the signal. 10 White noise is known to affect the auditory cortex, interfering, for example, with lexical decision making. 15 It is believed that the more similar the masker is to the signal, the greater the masking effect. However, studies have shown that white noise is capable of significantly affecting both P1, N1 and P2 latencies and amplitudes and N1-P2 peak-to-peak amplitude of CAEP with speech stimuli. 10 15 .

The analysis of the N1-P2 peak-to-peak amplitude appears to be important in some studies, as no significant difference can be found for each of the waves' amplitudes separately 7 16 . Their decrease or increase also reflect the number of recruited neurons, extent of neuronal activation, and neural synchrony involved in the response 7 9 16 .

Most studies on this topic have focused on ipsilateral simultaneous masking 8 9 10 11 12 13 14 15 . In this sense, the present study aims to analyze the effect of simultaneous contralateral white noise masking on CAEP with speech stimuli. For this end, CAEPs were recorded from the syllables /ba/ and /da/ in silence and with contralateral white noise for the analysis of latencies and amplitudes of waves P1, N1, and P2, in addition to N1-P2 peak-to-peak amplitude.

## Methodology

This is a cross-sectional observational analytical study carried out in a laboratory specialized in hearing and technology and was approved by the Research Ethics Committee of a public educational institution in the state of Alagoas under number 3,985,087.

The sample consisted of 15 participants of both sexes aged between 21 and 39 years old selected by convenience according to the established inclusion and exclusion criteria.

Participants with hearing thresholds up to 25 dB HL at frequencies from 250 to 8,000 Hz, external auditory canal free of obstructions, type “A” tympanometric curve, presence of acoustic reflexes and brainstem auditory evoked potential (BAEP) for neurodiagnosis without changes were included. Participants with changes in the outer and/or middle ear, exposure to occupational or leisure noise, otologic surgeries, more than three ear infections in the current year, use of ototoxic medication, cognitive changes, complaints of tinnitus, vertigo, dizziness or other cochleovestibular changes and complaints related to central auditory processing disorder were excluded.

The data collection procedures were divided into precollection procedures and procedure. The precollection procedures included:

1. Calibration of all equipment to be used;
2. Signature of the Free and Informed Consent Form;
3. Detailed anamnesis to investigate the pre-established inclusion and exclusion criteria;
4. Otoscopy to assess the external auditory canal and the tympanic membrane using the Heine mini 3000 otoscope;
5. Acoustic immittance measurements to select participants with type “A” tympanometric curve and detect acoustic reflexes using the middle ear analyzer Interacoustics 16 AT 235;
6. Pure tone audiometry to select participants with hearing thresholds up to 25 dB HL using the Interacoustics AD 629 audiometer, model DD45 supra-aural headphones and Vibrasom acoustic booth. Frequencies with octave ratios between 250 and 8,000 Hz, in addition to inter-octave frequencies of 3,000 and 6,000 Hz were assessed;
7. ABR with click stimulus, 10 ms recording window, speed of 21.1 stimuli/second, EEG bandpass filter from 100 to 3,000 Hz, gain of 100.0 K and duration of 100 μsec. Two recordings were performed with 2,000 stimuli, rarefied polarity and 80 dB dBnHL intensity. In the analysis of the tracings, latencies, morphology, and reproducibility of waves I, III and V and interpeak intervals I-III, III-V, and I-V were observed. Results with latency increase above two standard deviations and/or absence of any of the peaks were considered changed. The Biologic Navigator PRO was used.

The procedure was:

1. Cortical auditory evoked potentials with speech stimulus in two test conditions: 1) without noise; 2) with white noise at 100 dBSPL of contralateral intensity and simultaneous to the speech stimulus.

To perform the CAEP, the participants were positioned in a comfortable armchair, in an electrically-shielded and sound-attenuated booth, and instructed to remain relaxed and awake. They were instructed to remain alert during the assessment and, for this, were invited to watch short silent films, in black and white, during the test. The electrode region was prepared with an abrasive gel. Then, disk-type electrodes were placed with a conductive paste in the following positions: positive electrode at Cz (vertex); negative electrode at M2 (right mastoid); ground electrode at Fpz (forehead). For the condition without noise, stimuli were presented monaurally in the right ear. For the masking condition, the stimuli were presented in the right ear, and the noise was presented contralaterally in the left ear. All stimuli were presented via insert phones (EAR-phones 3A). The electrode impedance values, alone, were ≤3 KΩ and the difference between them was equal to 1 KΩ.

The 200-stimuli presentation followed the oddball paradigm, a proportion of 20% infrequent stimuli (syllable /da/) and 80% frequent stimuli (syllable /ba/), with 80 dBnHL intensity, speed of 0 .7 stimuli/second, alternating polarity and 1 to 30Hz bandpass filter. Two recordings were carried out and the analysis was performed on the tracing resulting from their weighted sums. The used equipment was the Navigator PRO - Biologic.

**Fig. 1:**
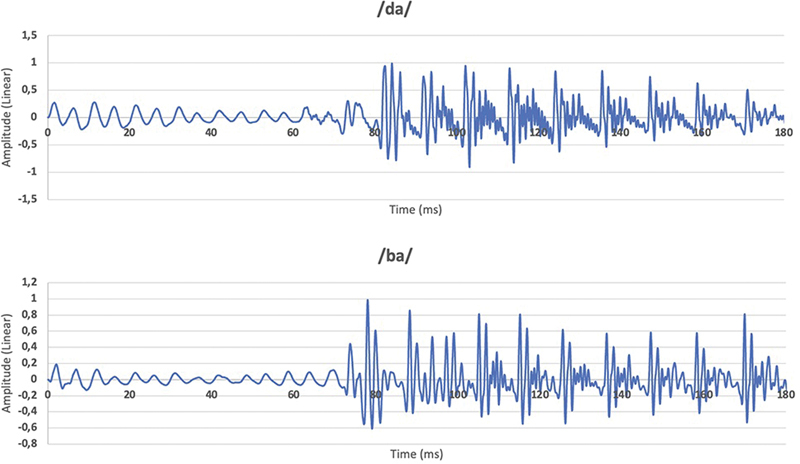
Spectrum of speech stimuli /da/ and /ba/ in the time domain.

**Fig. 2:**
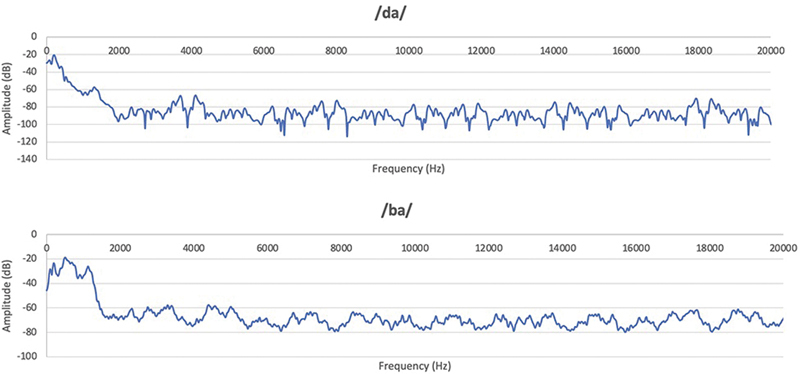
Spectrum of speech stimuli /da/ and /ba/ in the domain of frequencies.

The sampling rate of the speech stimuli used was 48 kHz and the resolution was 24 bits, both with a duration of 180 ms. The acoustic spectrum in the time domain and in the frequency domain can be found below, for each of them ( Figures 1 and 2 ). The speech stimulus /da/ has a fundamental frequency of 87.2 Hz while the stimulus /ba/ has a fundamental frequency of 91.8 Hz.

The CAEPs were recorded by an experienced examiner, and the P1, N1, and P2 wave markings were performed by at least two electrophysiology expert researchers. When the tracing was found challenging to analyze, and disagreement regarding the marking was verified, all professionals involved in the study discussed until consensus was reached. Each wave marking and identification was performed manually to assess its morphological characteristics and relevant temporal aspects.

Statistical analysis was performed using the IBM SPSS Statistics for Windows version 24.0 (IBM Corp., Armonk, NY, USA). Data description used tabular presentation of means, standard deviations (SDs) and confidence intervals (CIs). Initially, a sample assessment was carried out to observe its adherence to the normal distribution using the Shapiro-Wilk test. Then, to compare the pairs of test conditions, the Student t test or the Wilcoxon test were used, depending on the sample normality. Differences were considered significant when p values were < 0.05.

## Results

Fifteen individuals participated in the present study, 7 male and 8 females. Age ranged between 21 and 39 years old, with a mean of 27.13 years old, SD of 6.42 years, and lower CIs of 23.58 years and higher of 30.69 years.

**Fig. 3:**
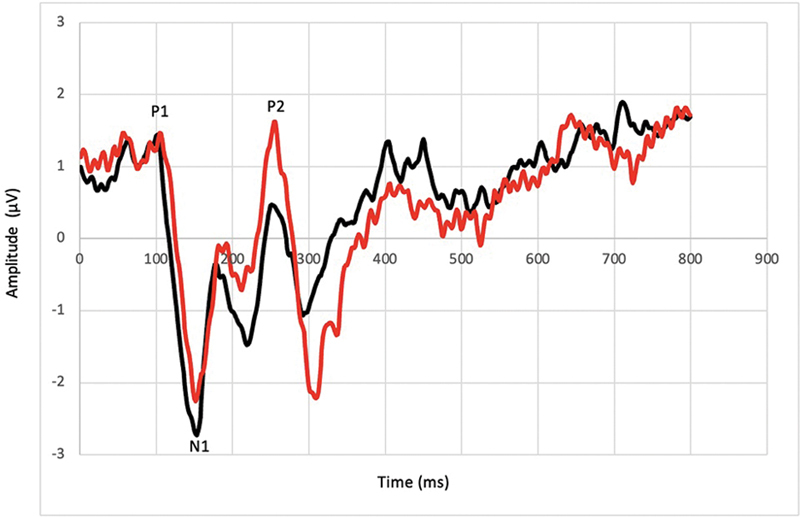
Grand average of the Cortical Auditory Evoked Potential waves in the conditions without noise and with contralateral white noise.

Figure 3 shows the grand average of CAEP with speech stimulus in the conditions without noise and with contralateral white noise at 100 dBSPL.

Table 1 shows the descriptive analysis (means, SDs, and CIs) of the P1, N1 and P2 latency and amplitude values and N1-P2 peak to peak amplitude for the two CAEP test conditions. The mean latency values of all waves increased for the noisy condition. The mean amplitude values of waves P1 and P2 and N1-P2 peak to peak amplitude also increased for the noisy condition, unlike the mean amplitude value of wave N1, which decreased.

**Table 1:** Descriptive analysis of P1, N1, and P2 latency and amplitude values for the two cortical auditory evoked potential test conditions (without noise and with contralateral noise)

Condition	Wave	Variable	Mean	SD	Lower CI	Upper CI
Without Noise	P1	Latency (ms)	53.13	6.70	49.42	56.84
		Amplitude (µv)	0.02	2.27	- 1.24	1.27
	N1	Latency (ms)	103.52	30.12	86.84	120.20
		Amplitude (µv)	- 3.83	1.95	- 4.91	- 2.75
	P2	Latency (ms)	157.65	37.63	136.81	178.49
		Amplitude (µv)	- 0.16	1.85	- 1.19	0.86
	N1-P2	Peak to peak amplitude (µv)	3.67	1.63	2.77	4.57
With Noise	P1	Latency (ms)	59.38	9.97	53.86	64.89
		Amplitude (µv)	0.58	1.51	- 0.26	1.41
	N1	Latency (ms)	109.62	25.50	95.50	123.75
		Amplitude (µv)	- 3.12	2.30	- 4.39	- 1.85
	P2	Latency (ms)	173.33	33.10	155.00	191.67
		Amplitude (µv)	0.88	2.42	-0.46	2.22
	N1-P2	Peak to peak amplitude (µv)	4.00	2.27	2.74	5.25

Abbreviations: CI, confidence interval; ms, milliseconds; SD, standard deviation; µv, microvolts.

Table 2 shows the comparative analysis of P1, N1, and P2 latency and amplitude values between the two CAEP test conditions. There was a significant difference between the two test conditions for the mean latency values of waves P1 and P2. Regarding the mean amplitude values, there was a significant difference for the P2 wave.

**Table 2:** Comparative analysis of P1, N1, and P2 latency and amplitude values between the two cortical auditory evoked potential test conditions (without noise and with contralateral noise) in the 15 participants

Wave	*p-value*	
	Amplitude	Latency
P1	0.343 a	0.047 a
N1	0.150 a	0.167 ^b^
P2	0.018 a *	0.013 ^b^ *
N1-P2	0.461 ^b^	−

a Student T statistical test, ^b^ Wilcoxon statistical test; *, p < 0.05.

## Discussion

On a daily basis, individuals are exposed to numerous sound stimuli simultaneously coming from different places in space. These sound stimuli interact with each other, challenging the understanding of a target stimulus, such as speech. This is a complex task for individuals with normal hearing thresholds and becomes even harder for individuals with hearing loss and for the elderly. 17 18

Such difficulty can be explained by the phenomenon of auditory masking and is observed in electrophysiological test records. 3 6 In this context, the present study identified changes in the morphology of the CAEP recordings. When white noise was presented contralaterally and simultaneously to CAEP with speech stimulus, the P1, N1, and P2 latencies increased ( Table 1 ). The amplitudes of P1 and P2 also increased, unlike the amplitude of N1, which decreased ( Table 1 ).

The differences were significant for the P1 and P2 wave latencies for the P2 wave amplitude ( Table 2 ), revealing a simultaneous masking effect by the contralateral white noise in the studied population. Although the difference was not significant for the N1 wave latency, there was a pattern of increase for all wave latencies ( Table 1 ). Latency is considered to be more affected by auditory masking in comparison with amplitude measures. 19

However, auditory masking is also expected to affect the CAEP amplitude, decreasing it. 9 10 11 12 13 14 In the present study, although the difference was not significant, the N1 amplitude decreased ( Table 1 ) when white noise was presented contralaterally and simultaneously with CAEP with speech stimulus. The increase in P1amplitudes, with no significant difference, and in P2, with a significant difference ( Table 2 ), can be explained as the central masking effect is considered smaller in relation to peripheral masking. 20

Central masking occurs in dichotic listening when signal and masker are presented separately in each ear, while peripheral masking occurs in monotic listening when signal and masker are presented in the same ear. The effect of peripheral masking becomes greater, since the spectral overlap between signal and masker occurs from the cochlea. 20

In addition to these two types of masking, there is the informational masking. Auditory masking is even greater when signal and masker are similar. For example, speech comprehension is strongly disturbed by simultaneous speakers, since multiple interferences at the acoustic, phonological, and semantic levels occur. 21 It is noteworthy that white noise also has strong interference in the neural processing of speech. 15

The analysis of P1, N1, and P2 waves was performed from the recordings obtained in the right ear. This is due to the fact that the left cerebral hemisphere is considered responsible for decoding linguistic sounds related to speech and language. After the decussation of the pyramidal tracts, the crossing of auditory information from each ear occurs. 22 For this reason, during simultaneous masking, speech stimuli (/ba/ and /da/) were presented to the right ear and white noise was presented to the left ear.

The theme, specifically from the CAEP recording perspective, is still insufficiently discussed in the literature. In addition, standard protocols for assessing auditory masking based on the CAEPs have not been documented yet. Thus, the present study represents a new tool for the analysis of the effect of simultaneous masking in CAEPs elicited by speech stimuli. Moreover, we expect to contribute to further studies and to the development of new technologies and preventive, interventionist, and follow-up actions for patients with complaints of difficulties in speech-in-noise perception. On a daily basis, individuals are exposed to numerous sound stimuli simultaneously coming from different places in space. These sound stimuli interact with each other, challenging the understanding of a target stimulus, such as speech. This is a complex task for individuals with normal hearing thresholds and becomes even harder for individuals with hearing loss and for the elderly. 18 19

Such difficulty can be explained by the phenomenon of auditory masking and observed in electrophysiological test records. 3 6 In this context, the present study identified changes in the morphology of the CAEP recordings. When white noise was presented contralaterally and simultaneously to CAEP with speech stimulus, the P1, N1, and P2 latencies increased ( Table 1 ). The amplitudes of P1 and P2 and N1-P2 peak to peak amplitude also increased, unlike the amplitude of N1, which decreased ( Table 1 ).

The differences were significant for the P1 and P2 wave latencies for the P2 wave amplitude ( Table 2 ), revealing a simultaneous masking effect by the contralateral white noise in the studied population. Although the difference was not significant for the N1 wave latency, there was a pattern of increase for all wave latencies ( Table 1 ). Latency is considered to be more affected by auditory masking in comparison with amplitude measures. 20

CAEP responses are recorded without the active participation of the examined individual. Therefore, waves P1, N1, and P2 reflect the initial phases of neural speech processing and are affected by the acoustic characteristics of the stimulus. Once the noise is associated with the stimulus, these characteristics change, eliciting responses with reduced temporal precision 21 . The increase in N1 latency found not significant in this study can be attributed to the reduced sample size. However, auditory masking is also expected to affect the CAEP amplitude, decreasing it. 9 10 11 12 13 14 15 16 In the present study, although the difference was not significant, the N1 amplitude decreased ( Table 1 ) when white noise was presented contralaterally and simultaneously with CAEP with speech stimulus. The increase in P1 amplitudes and N1-P2 peak to peak, with no significant difference, in addition to P2, with a significant difference ( Table 2 ), can be explained as the central masking effect is considered smaller in relation to peripheral masking already analyzed in previous studies 22 .

Central masking occurs in dichotic listening when signal and masker are presented separately in each ear, while peripheral masking occurs in monotic listening when signal and masker are presented in the same ear. The effect of peripheral masking becomes greater, since the spectral overlap between signal and masker occurs from the cochlea. 22

In addition to these two types of masking, there is the informational masking. Auditory masking is even greater when signal and masker are similar. For example, speech comprehension is strongly disturbed by simultaneous speakers, since multiple interferences at the acoustic, phonological and, semantic levels occur. 23 It is noteworthy that white noise also has strong interference in the neural processing of speech. 15 A study with neuroimaging showed that this noise affects the auditory cortex, interfering, for instance, with lexical decision making 24 .

Another factor that may explain the increase in P1, P2 and N1-P2 peak-to-peak amplitudes in this study was that the intensity of the contralateral white noise was lower (100 dBSPL) in comparison with the speech stimulus intensity (80 dBnHL). Studies with simultaneous ipsilateral white noise have shown that the masking effect is smaller for positive signal-to-noise ratios, that is, when the signal is presented with greater intensity in relation to the masking. These studies also showed that there is no rigid pattern of amplitude decrease or latency increase as the masker is presented with greater intensity in relation to the signal, and there may be an increase in amplitude and decrease in latency 10 15 .

The analysis of P1, N1, and P2 waves was performed from the recordings obtained in the right ear. This is due to the fact that the left cerebral hemisphere is considered responsible for decoding linguistic sounds related to speech and language. After the decussation of the pyramidal tracts, the crossing of auditory information from each ear occurs. 25 For this reason, during simultaneous masking, speech stimuli (/ba/ and /da/) were presented to the right ear and white noise was presented to the left ear.

The theme, specifically from the CAEP recording perspective, is still insufficiently discussed in the literature. In addition, standard protocols for assessing auditory masking based on the CAEP have not been documented yet. Thus, the present study represents a new tool for the analysis of the effect of simultaneous masking in CAEP elicited by speech stimuli. Moreover, we expect to contribute to further studies and to the development of new protocols and preventive, interventionist and follow-up actions for patients with complaints of difficulties in speech-in-noise perception.

## Conclusion

The effect of simultaneous masking was observed from the changes in the morphology of the CAEPs elicited by speech stimulus when white noise was presented in the contralateral ear. There was a significant increase in P1 and P2 wave latencies, as well as in P2 wave amplitude.
